# Variability of blood eosinophil count and prognosis of COPD exacerbations

**DOI:** 10.1080/07853890.2021.1949489

**Published:** 2021-07-16

**Authors:** Sandra Martínez-Gestoso, María-Teresa García-Sanz, Uxío Calvo-Álvarez, Liliana Doval-Oubiña, Sandra Camba-Matos, Francisco-Javier Salgado, Xavier Muñoz, Purificación Perez-Lopez-Corona, Francisco-Javier González-Barcala

**Affiliations:** a Emergency Department, Salnés County Hospital, Vilagarcía de Arousa, Spain; b Respiratory Medicine Department, Arquitecto Marcide Hospital, Ferrol, Spain; c Respiratory Medicine Department, University Hospital Complex of Santiago de Compostela, A Coruña, Spain; d Respiratory Medicine Department, Hospital Vall d’Hebron, Barcelona, Spain

**Keywords:** COPD, exacerbation, prognosis, eosinophilia

## Abstract

**Background:**

Eosinophils in peripheral blood are one of the emerging biomarkers in chronic obstructive pulmonary disease (COPD) patients. However, when analysing the relationship between peripheral eosinophilia and COPD prognosis, highly variable results are obtained. The aim of our study is to describe the serum eosinophilia levels in COPD patients and to analyse their relationship to prognosis following hospital admission.

**Methods:**

A prospective observational study was conducted from 1 October 2016 to 1 October 2018 in the following Spanish centres: Salnés County Hospital in Vilagarcía de Arousa, Arquitecto Marcide Hospital in Ferrol and the University Hospital Complex in Santiago de Compostela. The patients were classified using three cut-off points of blood eosinophil count (BEC): 150 cells/µL, 300 cells/µL, and 400 cells/µL; in addition, the peripheral BEC was analysed on admission.

**Results:**

615 patients were included in the study, 86.2% male, mean age 73.9 years, and mean FEV1 52.7%. The mean stay was 8.4 days, and 6% of all patients were readmitted early. No significant relationship was observed between the BEC, neither in the stable phase nor in the acute phase, and hospital stay, readmissions, deaths during admission, the need for intensive care, or the condition of frequent exacerbator.

**Conclusion:**

The results of our study do not seem to support the usefulness of BEC as a COPD biomarker.

**KEY MESSAGES**
1. There is evidence that BEC participates in pathophysiological mechanisms of the COPD.
2. BEC may be useful as a biomarker in COPD for aspects such as the optimization of treatments.
3. We did not find any relationship between BEC levels and prognosis following hospital admission for AECOPD.

## Introduction

Chronic obstructive pulmonary disease (COPD) is a heterogeneous entity with tobacco smoking as the main aetiological factor; environmental pollutants or patient characteristics, such as alpha-1 antitrypsin deficiency, are also frequently involved [1,2]. Exacerbations are particularly relevant in the course of the disease, as they account for the largest share of COPD-related costs [1]. They are also a good indicator of a worse prognosis since COPD mortality rate is 26.2% in the first year and 64.3% at 5 years [3] following an exacerbation requiring hospital admission. In addition, COPD patients frequently show comorbidities such as bronchiectasis, arterial hypertension, diabetes or heart failure, which interfere with both the diagnosis and the therapeutic management [4–6].

At present, patient-tailored treatments are available, improving the disease prognosis [7–9]. Personalized care, based on the individualized management of patients based on their clinical characteristics and certain biological factors, makes it possible to maximize the benefits and minimize the side effects at a reasonable cost [10,11]. In recent years, eosinophils in peripheral blood are one of the emerging biomarkers in patients with COPD, since there is evidence that they participate in pathophysiological mechanisms of the disease and they are also easily accessible since they are available in most laboratories [12–15].

When analysing the relationship between the peripheral blood eosinophil count (BEC) and the prognosis of COPD, highly variable results are obtained. While some authors do not observe any relationship between BEC and the incidence of exacerbations [14,16–20], others find a higher incidence of exacerbations with a higher BEC level [21–23]. Regarding mortality, discrepancies are also observed: some authors note a lower mortality with an increasing BEC [24,25], while no significant relationship is found in other studies [26,27].

Another controversial aspect is the stability in BEC values over time since a single estimate of the BEC count does not seem to adequately reflect the cell pattern in a given patient. A 3-year follow-up study with ECLIPSE patients showed that only 51% of them remained stable above or below a defined BEC cut-off point [28]. Other authors refer greater stability, with 69.3% of the patients having similar BEC levels over a year, as reported in the BODE and CHAIN cohorts [29,30].

The aim of our study was to describe the levels of serum eosinophilia in patients with COPD, to analyse the clinical characteristics of these patients and their prognosis following hospital admission, based on their BEC levels.

## Methods

Prospective observational study conducted from 1 October 2016 to 1 October 2018 in the following Spanish centres: Salnés County Hospital in Vilagarcía de Arousa, Arquitecto Marcide Hospital in Ferrol and the University Hospital Complex in Santiago de Compostela. Patients admitted for acute exacerbations of COPD (AECOPD) who agreed to participate and signed informed consent were included. Exclusion criteria were as follows: admission for non-AECOPD condition, severe cognitive impairment and dementia.

The diagnosis, baseline severity, and AECOPD were defined following the GOLD criteria [1]. The patients without available baseline spirometry included in the study were diagnosed with COPD based on clinical, radiological and epidemiological criteria (previous compatible symptoms, chest X-ray suggestive of COPD and history of smoking) by the specialist responsible for admission, and subsequently reviewed by two pulmonologists from the research team [31]. Baseline dyspnoea was classified as per the eMRCD scale [1]. Comorbidity was assessed with the Charlson Index [32]. Patients who had not smoked for more than a year at the time of admission were considered former smokers [33]. The body mass index (BMI) was categorized into four groups, following World Health Organization (WHO) criteria: underweight (BMI < 18.5); normal weight (18.5 ≤ BMI < 25); overweight (25 ≤ BMI < 30); and obesity (BMI ≥ 30) [34]. The baseline treatment of the patients was identified from the clinical history. The use of at least 5 mg of prednisone for 3+ consecutive months was considered chronic steroid treatment [35]. Vital signs, arterial blood gases and chest X-rays were obtained upon arrival of the patient at the emergency department (ED). Blood count and serum biochemistry data were recorded in the ED and in the hospital ward.

The patients were classified as persistently low, persistently high, or intermittent based on the results of 3 BEC records with at least a 3-month gap, including those obtained in the previous year and in the year after the index hospital admission. Those patients with all 3 BEC records above the predefined cut-off point were classified as persistently high; those with all 3 records below the cut-off point were classified as persistently low; and those with records both above and below the cut-off point were classified as intermittent. The cut-off points used were 150 cells/µL, 300 cells/µL and 400 cells/µL. In addition, the BEC at the time of admission for COPD was analysed. Those patients with a stay equal to or longer than the median stay of the study population were identified as patients with prolonged admission [36]. Early readmission was defined as that occurring within the first 15 days following discharge for AECOPD [37]. Those patients with two or more exacerbations in the previous year were considered frequent exacerbators [22].

All patients included signed the informed consent and the study was approved by the Galician Ethical Committee (Registry Code 2016/460).

### Statistical analysis

Data obtained by statistical analysis are expressed as mean ± SD in continuous variables and as frequencies and percentages in categorical variables. Continuous variables were compared using the Student’s *t*-test or the Wilcoxon test; in the case of categorical variables, the chi-square test and the Fisher’s exact test were used. A multivariate analysis using logistic regression was planned, including those variables with *p* ≤ .05 in the univariate analysis, but it was eventually discarded as no significant results were obtained in the univariate analysis. Variables associated with a *p* < .05 were considered statistically significant. Analyses were carried out with SPSS 15.

## Results

615 patients were included in the study, 86.2% male, mean age 73.9 years, and mean FEV1 52.7%. The mean stay was 8.4 days, and 6% of all patients were readmitted early A total of 95 patients (15.4%) required non-invasive ventilation, 9 of them at the ICU. 80 patients had acute respiratory acidaemia; 60% of them required non-invasive ventilation. Hospital mortality was 3.7% (Table 1).

**Table 1.** Characteristics of the study population (*n* = 615).

Male (%)	530 (86.2%)
Mean age	73.9 (10.6)
Smoker status	
Unknown	23 (3.7%)
Active	163 (26.5%)
Former smoker	383 (62.3%)
Never smoker	46 (7.5%)
BMI	
Underweight	17 (3.4%)
Normal weight	114 (22.8%)
Overweight	202 (40.4%)
Obesity	167 (33.4%)
FEV1%, mean (SD)	52.7 (18.9)
GOLD	
Mild	54 (8.4%)
Moderate	217 (35.3%)
Severe	136 (22.1%)
Very severe	148 (24.1%)
Unclassifiable	60 (9.8%)
Anthonisen (no. of criteria)	
0	16 (2.6%)
1	223 (36.3%)
2	165 (26.8%)
3	211 (34.3%)
Charlson (points)	
0	2 (0.3%)
1, 2	96 (15.7%)
>2	513 (84%)
Admissions previous year	
0	350 (56.9%)
1	135 (22%)
≥2	130 (21.1%)
ED previous year	
0	305 (49.6%)
1	142 (23.1%)
≥2	168 (27.3%)
Baseline treatment	
SABA	252 (41%)
SAMA	131 (21.3%)
LABA	479 (77.9%)
LAMA	406 (66%)
ICS	393 (63.9%)
OCS	36 (5.9%)
Theophylline	63 (10.2%)
Azithromycin	21 (3.4%)
Home oxygen	148 (24.1%)
Systemic corticosteroid during admission	547 (88.9%)
Mean stay (days)	8.4 (6.2)
Prolonged stay (P50)	336 (54.6%)
Prolonged stay (P75)	145 (23.6%)
Readmission at 15 days	37 (6%)
Readmission at 30 days	104 (16.9%)
Death during admission	23 (3.7%)
ICU	15 (2.4%)
NIV	95 (15.4%)

BMI: body mass index; ED: emergency department; ICS: inhaled corticosteroids; ICU: intensive care unit; LABA: long-acting beta agonists; LAMA: long-acting anticholinergic agonist; NIV: non-invasive mechanical ventilation; OCS: oral corticosteroids; SABA: short-acting beta agonists; SAMA: short-acting anticholinergic agonists.

**Figure 1.**
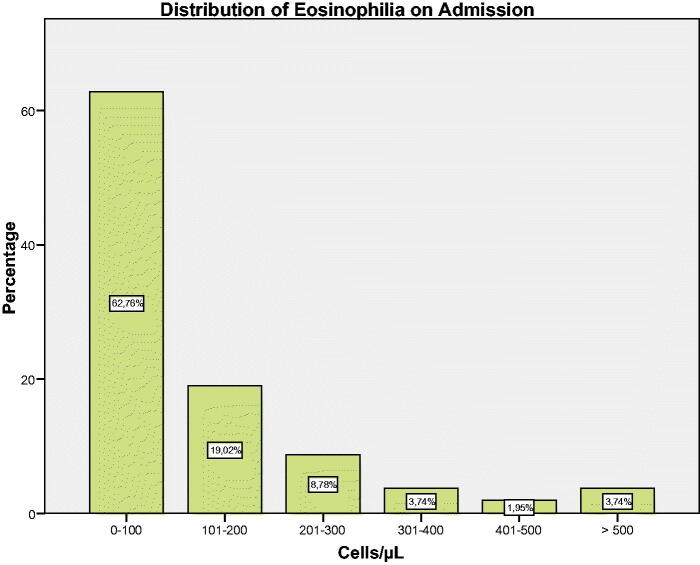
Distribution of eosinophilia on admission.

On admission, 63.4% of all patients showed ≤100 cells/µL (Figure 1). In the stable phase, 43.3% of all patients met persistently high criteria with a cut-off point of 150 cells/µL, 14.3% with a cut-off point of 300 cells/µL, and 5.6% with a cut-off point of 400 cells/µL. The intermittent BEC criterion was observed in 39.5%, 33.2% and 21% respectively (Table 2). The characteristics of the study population did not differ when performing subgroup analyses based on baseline BEC and BEC at the time of exacerbation (data not shown).

**Table 2.** Prognosis of COPD exacerbation in relation to baseline BEC.

	Persistently high 150	Intermittent	Persistently low	*p* Value
	265 (43.3%)	242 (39.5%)	105 (17.2%)	
Mean stay (days)	8.4 (6.6)	8.5 (5.9)	8.2 (5.5)	.906
Stay > p75	55 (20.8%)	64 (26.4%)	25 (23.8%)	.319
Readmission at 15 days	14 (5.3%)	18 (7.4%)	5 (4.8%)	.496
Readmission at 30 days	33 (12.5%)	53 (21.9%)	18 (17.1%)	.018
Death during admission	12 (4.5%)	7 (2.9%)	4 (3.8%)	.626
ICU	6 (2.3%)	7 (2.9%)	2 (1.9%)	.832
Frequent exacerbator				.895
≥2 admissions/ED previous year	90 (43.5%)	79 (38.2%)	38 (18.4%)	
None	104 (43%)	95 (39.3%)	43 (17.8%)	

	Persistently high 300	Intermittent	Persistently low	*p*
	88 (14.3%)	204 (33.2%)	323 (52.5%)	
Mean stay (days)	7.8 (4.6)	8.3 (5.2)	8.6 (7.0)	.543
Stay > p75	17 (19.3%)	51 (25%)	77 (23.8%) 0.569	
Readmission at 15 days	3 (3.4%)	18 (8.8%)	16 (5%)	.103
Readmission at 30 days	12 (13.6%)	32 (15.7%)	60 (18.6%) 0.466	
Death during admission	2 (2.3%)	7 (3.4%)	14 (4.3%)	.639
ICU	1 (1.1%)	3 (1.5%)	11 (3.4%)	.259
Frequent exacerbator				.133
≥2 admissions/ED previous year	39 (18.8%)	62 (30%)	106 (51.2%)	
None	29 (11.8%)	80 (32.7%)	136 (55.5%)	

	Persistently high 400	Intermittent	Persistently low	*p*
	34 (5.5%)	129 (21%)	452 (73.5%)	
Mean stay (days)	7.1 (4.2)	8.2 (4.9)	8.6 (6.6)	.374
Stay > p75	5 (14.7%)	31 (24%)	109 (24.1%)	.456
Readmission at 15 days	1 (2.9%)	10 (7.8%)	26 (5.8%)	.519
Readmission at 30 days	4 (11.8%)	23 (17.8%)	77 (17%)	.697
Death during admission	2 (5.9%)	2 (1.6%)	19 (4.2%)	.298
ICU	0 (0%)	2 (1.6%)	13 (2.9%)	.440
Frequent exacerbator				.276
≥2 admissions/ED previous year	16 (7.7%)	48 (23.2%)	143 (69.1%)	
None	11 (4.5%)	45 (18.4%)	189 (77.1%)	

ED: emergency department; ICU: intensive care unit.

No significant relationship between BEC and hospital stay, readmissions, deaths during admission, the need for intensive care or the condition of frequent exacerbator was observed, in neither the stable nor the acute phase (Table 2).

## Discussion

Our study confirms a significant variability in the BEC of patients with COPD over time. Although the majority remained in the same group over all 3 measurements, 40.4% of all patients at a BEC cut-off point of 150 cells/µL and 34.3% at a BEC cut-off point of 300 cells/µL changed groups. These data are similar to those reported by Casanova et al., with the same cut-off point of 300 cells/µL and 3 measurements, where 43.9% of all patients in the CHAIN cohort and 30.2% in the BODE cohort change groups during follow-up [30]. Other authors obtain different rates of patients changing groups over time, ranging 16.4%–65%. However, it is difficult to establish comparisons since they use different methodologies, with different cut-off points or a different number of BEC records [14,15,23,28,29,38]. We wonder whether this known BEC variability could be one of the factors behind the discrepancy across the different studies, in which case establishing persistently high or persistently low groups could be a better indicator of the eosinophilic phenotype and could clarify the relationship between the BEC and the prognosis of COPD. However, no relationship between the BEC and the prognosis of patients with COPD was observed in any of the indicators evaluated in our population.

There appears to be a significant difference in readmission at 30 days in the 150 cell/µL cut-off group. However, this association disappears when performing a multivariate analysis adjusted for other variables, regardless of corticosteroid treatment and hospitalization for exacerbation in the previous year, which was similar in all 3 groups. Singh et al. also found no solid association between BEC and the risk of AECOPD: they reported a trend towards lower rates of AECOPD in patients with ≤150 cells/µL, although their results may have been impacted by the use of corticosteroids. They did consider previous exacerbations as the best predictor of future exacerbations, which is consistent with the existing literature [39].

Other papers assessing BEC with at least two records show contradictory results. Some authors agree with our results and do not observe differences in the rate of exacerbations based on the level of BEC [14,25,28,30,38].

However, other researchers report a higher risk of exacerbations in patients with a persistent ≥300 cells/µL BEC [23,40], and a higher survival in patients with persistently high BEC compared to a persistently low or intermittent BEC [25,30,38]. We have found a single paper analysing the stability of BEC levels and hospital stay, reported to be shorter for predominantly eosinophilic patients [25]. The results reported by other authors, who observed a U-shape distribution in the risk of exacerbations, make it more difficult to correctly interpret the BEC value as a prognostic biomarker in COPD. Vedel-Krogh et al. observed more exacerbations in the groups below 130 cells/µL and in those above 340 cells/µL [41], an observation similar to that by Miravitlles et al., with a higher number of exacerbations in patients below 150 cells/µL and in those above 500 cells/µL [42]. At least in part, the discrepancies in the results observed may be due to methodological differences that are relevant in some cases, such as the mean age of the patients included (62–71 years), the baseline FEV1 (48%–68%), the percentage of male patients (51%–85%), and the cut-off points, which is the BEC absolute value in some cases and the percentage in others [14,30,38,40].

The discrepancies across the different studies could be related to the fact that the BEC is not useful as a prognostic indicator in COPD, or that it is useful only in some patient subgroups. Thus, a BEC above 300 has been recently reported as a good predictor of future exacerbations in patients with two or more exacerbations in the previous year, but not in patients without exacerbations [40]. However, this finding has not been solidly established in the literature either, as the opposite result has been reported in a recent meta-analysis, linking a high BEC to a higher risk of exacerbations in those patients with no hospital admissions in the previous year [43]. Finally, other aspects to consider when interpreting the usefulness of BEC as a prognostic factor in COPD would include the correlation between the BEC and the activity of eosinophils in the respiratory system, the measurement method, which may differ across laboratories; and the treatments performed, whose response may differ depending on the levels of BEC [13].

Limitations of this study include the fact that COPD severity and spirometry results were not available in 20% of all patients, and diagnosis of COPD was established on the basis of symptoms, chest X-ray and history of smoking upon review by the pulmonologists in the research team. Even though these criteria were accepted in previous studies, they may have affected the results. The patients in this study followed their baseline treatment and received medication to control exacerbations as decided by their doctors, so we cannot rule out that the results obtained may have been affected by an indication bias.

Although this study was not designed to assess the response to steroid treatment, the high proportion of patients requiring inhaled corticosteroids (around 64%) or systemic corticosteroids during admission (88.9%) may have affected the results. However, the patients in the subgroups analysed according to the BEC did not show significant differences in relation to the use of corticosteroids.

Although the BEC may be useful as a biomarker in some patient subgroups or for aspects such as the optimization of some treatments, the results of our study do not seem to support this, as they do not allow to consider the BEC as a predictor of prognosis, including future exacerbations or previous history of admission for AECOPD.

In conclusion, we did not find any relationship between BEC levels and prognosis following hospital admission for AECOPD. The differences in methodology and results across the different studies seem to indicate the need for further research in this field.

## Data Availability

The data that support the findings of this study are available from the corresponding author, M.T.G.S., upon reasonable request.
